# Clinical Factors for Predicting Pharmacotherapy in Twin Pregnancies Complicated by Gestational Diabetes Mellitus

**DOI:** 10.3390/jcm12216856

**Published:** 2023-10-30

**Authors:** Anja Catic, Theresa Reischer, Florian Heinzl, Christian Göbl, Gülen Yerlikaya-Schatten

**Affiliations:** 1 Department of Obstetrics and Gynecology, Division of Feto-Maternal Medicine, Medical University of Vienna, 1090 Vienna, Austria; anja.catic@meduniwien.ac.at (A.C.); florian.heinzl@meduniwien.ac.at (F.H.); christian.goebl@meduniwien.ac.at (C.G.); guelen.yerlikaya-schatten@meduniwien.ac.at (G.Y.-S.); 2 Fetal Medicine Unit, Liverpool Women’s Hospital, Liverpool L8 7SS, UK

**Keywords:** GDM, twin pregnancy, pharmacotherapy, prediction

## Abstract

Controlling blood glucose levels is the main target in pregnant women with gestational diabetes mellitus (GDM). Twin pregnancies are offered the same screening selection and have the same diagnostic criteria as well as treatment of gestational diabetes as singleton pregnancies, even though the risks for pregnancy complications are increased. The aim of this study was to assess the association between predicting factors, OGTT glucose levels and pharmacotherapy requirements in twin pregnancies with GDM. This retrospective cohort study included 446 GDM patients with twin pregnancies (246 managed with lifestyle modifications and 200 requiring pharmacotherapy) over a time period of 18 years. An evaluation of maternal characteristics and a standardized 75 g oral glucose test (OGGT) for glucose concentrations at fasting, 1 h and 2 h were conduced. OGTT glucose levels at fasting (=0 min, *p* < 0.01) and 1 h (*p* < 0.01) were significantly associated with the later requirement of pharmacotherapy. Also, clinical risk factors (pre-pregnancy BMI *p* < 0.01, multiparity *p* < 0.05, GDM in previous pregnancy *p* < 0.01, assisted reproduction *p* < 0.05) showed a predictive accuracy for insulin therapy in twin pregnancies complicated by GDM, whereas age and chorionicity had no effect. OGTT glucose measures in addition to clinical risk factors are promising variables for risk stratification in mothers with GDM and twin pregnancy.

## 1. Introduction

Gestational diabetes (GDM) is defined as glucose tolerance impairment, which is detected during pregnancy. It is diagnosed by a 75 g oral glucose tolerance test (OGTT) over 2 h. Thereby, formal systematic testing for gestational diabetes usually takes place between 24 and 28 weeks of gestation [1,2]. Women with hyperglycemia detected during pregnancy are at greater risk for perinatal morbidity and mortality, at higher risk for operative delivery and at higher risk to develop a type 2 diabetes mellitus [3,4] as well as cardiovascular disease postnatally [5]. Women with gestational diabetes and strict glycemic control have better pregnancy outcomes compared to females with GDM who do not receive treatment [6,7]. Additionally, the treatment of GDM reduces the likelihood of serious perinatal morbidity [6].

Twin pregnancies are offered the same screening options and underlie the same diagnostic criteria as well as treatment of GDM as singleton pregnancies, even though the risk for pregnancy complications such as stillbirth, preterm birth, fetal growth restriction and preeclampsia are increased [8,9,10]. However, it is important to note that studies examining twin pregnancies complicated by GDM have produced conflicting results regarding the risk of gestational diabetes in twin deliveries. As some studies have found, twin pregnancies are at higher risk for GDM [11,12,13,14], while others find similar rates of GDM [15,16]. Moreover, studies indicating that the risk for GDM in twin pregnancies is higher than in singleton pregnancies have found the odds for GDM to be 1.4 times and 2.8 times higher for dichorionic and monochorionic twin pregnancies, respectively, as compared to singleton pregnancies [17]. The relative risk for the development of GDM in twin pregnancies was 1.13 [14]. Studies that have demonstrated a comparable risk of gestational diabetes mellitus in singleton and twin pregnancies reported a prevalence of 3.4% for both types of pregnancies [15]. 

Multiple pregnancy is attributed to a higher maternal age at conception and the growing use of assisted reproductive treatments [18]. Controlling blood glucose levels is the primary goal in managing women with GDM. An individual treatment plan consisting of lifestyle modification (dietary recommendations, exercise, self-monitoring of blood glucose levels) and pharmacotherapy if indicated with insulin and/or metformin should be evaluated in each patient in order to ensure normal fetal development and better perinatal outcomes [6]. Studies show different risk factors associated with the requirement for glucose-lowering medications for glycemic control in pregnancies complicated by GDM such as age, pre-pregnancy BMI, prior history of GDM, glycated hemoglobin value at GDM diagnosis and family history of type 2 diabetes mellitus [19,20,21,22,23], as well as higher fasting and 2 h plasma glucose concentrations during OGTT [24,25] as strong indicators for insulin therapy. Previous studies have indicated that increased OGTT glucose concentrations were related to adverse gestational and fetal outcomes [26,27].

The aim of this study is to compare the characteristics of women who required insulin therapy with those who needed lifestyle intervention solely during their twin pregnancy complicated by GDM and to identify the factors predicting insulin need in patients with twin pregnancy.

## 2. Materials and Methods

### 2.1. Patient Population

This retrospective cohort study was conducted at our outpatient clinic of the Medical University of Vienna, Division of feto-maternal Medicine between January 2003 and December 2021. We included 446 pregnant women with twin pregnancies and gestational diabetes. Of these, 246 were managed with lifestyle modifications (LSMs) and 214 required pharmacotherapy with insulin (IT, *n* = 200), metformin (*n* = 6) or both (*n* = 8). 

The importance of addressing maternal hyperglycemia was clearly illustrated in the Hyperglycemia and Adverse Pregnancy Outcomes (HAPO) study [27]. This research unequivocally established the connection between maternal glucose levels and adverse pregnancy outcomes, which subsequently prompted the International Association of Diabetes and Pregnancy Groups (IADPSG) to introduce revised diagnostic criteria in 2010. According to these criteria, a diagnosis of gestational diabetes mellitus is established if there is at least one abnormal value: ≥92 mg/dL for fasting blood glucose levels, ≥180 mg/dL for the 1 h plasma glucose level and ≥153 mg/dL for the two-hour plasma glucose concentrations, respectively, following a 75 g OGTT [1,2]. Before 2010, the diagnosis for GDM at our site was defined as one pathological value: ≥95 mg/dL blood glucose levels at fasting, ≥180 mg/dL plasma glucose level at 1 h and ≥155 mg/dL plasma glucose concentrations at 2 h, respectively, according to the guidelines of the German Society for Diabetes (DDG) [28]. From 2003 to 2010, our clinic managed a total of 99 cases of twin pregnancies with GDM. Among these, 31 cases were monochorionic twins, while 68 cases were dichorionic twin pregnancies. After 2010 and until December 2021, our clinic provided care for a total of 347 twin pregnancies complicated by GDM. Within this cohort, 106 cases were related to monochorionic twins, while 241 were classified as dichorionic twin pregnancies. All cases included in this study met the diagnostic criteria of GDM at the time of their diagnosis. As the specific diagnostic threshold was not a determining factor for our analysis, we included all cases in the study, irrespective of when the diagnosis was made.

In high-risk pregnancies (especially those with a previous history of GDM) for gestational diabetes, OGTT was conducted at any time point earlier than between 24 and 28 weeks of gestation. Otherwise, OGTT was performed at the recommended standardized gestational age between 24 and 28 weeks of pregnancy in all women with previous non-pathological glucose metabolism. As Metformin remains a medication of off-label use in pregnancy and is lacking guidelines for its prescription, further evaluation of Metformin therapy was neglected.

All patients were instructed for autonomous capillary blood glucose monitoring and informed about glycemic treatment targets. Follow-ups within two-to-three weeks were scheduled and charts of blood glucose levels were reviewed during each visit alongside with biometric ultrasound measurements for percentile growth and amniotic fluid assessment until delivery. Lifestyle modifications including an individual treatment plan consisting of medical nutrition therapy and moderate exercise was used as the first-line intervention together with self-glucose monitoring. If target blood glucose levels were not achieved, a pharmacologic intervention, additionally to the lifestyle changes, was initiated at any point. This intervention was implemented based on specific glucose threshold values, with consideration of the following criteria: 1. Fasting glucose levels: pharmacological intervention was initiated when fasting blood glucose levels were consistently equal to or exceeded 94 mg/dL (≥94 mg/dL at fasting). 2. Post-prandial glucose levels: pharmacological treatment was considered when post-prandial glucose levels reached or exceeded 140 mg/dL (≥140 mg/dL) one or two hours after each meal [29]. All women were instructed to continue lifestyle modification, even if pharmacotherapy was initiated. 

Additionally, the following variables were collected from the medical records: age, gravidity, parity, pre-pregnancy body mass index (BMI), a previous history of GDM in a preceding pregnancy, chorionicity, and OGTT results at fasting (G0), 60 min (G60) and 120 min (G120). Patient records were electronically examined with the assistance of the obstetric database PIA Fetal Database software, Version 5.6.16.917, (ViewPoint, GE Healthcare^®^, Munich, Germany). Subsequently, the data were retrieved and used for a statistical assessment. Patients with preexisting diabetes or a newly diagnosed diabetes mellitus other than gestational diabetes or with missing OGTT glucose values and those undergoing therapy with Metformin were excluded from the study. Furthermore, multiple pregnancies with more than two fetuses were excluded. For our statistical evaluation, a standardized examination of HbA1c at the time of diagnosis for our patient collective was not available. This study was approved by the Ethics Committee of the Medical University of Vienna and performed in accordance with the Declaration of Helsinki.

### 2.2. Statistical Analysis 

Statistical analysis was performed with R, a statistical programming language, version 4.3.1 [30]. Several R packages were utilized to aid in data processing and visualization. These packages included: tidyverse v2.0.0 [31], viridis v0.6.3 [32], ggeffects v1.2.3 [33], flextable v0.9.2 [34] and Barnard v1.8. [35]. Numerical variables and ordinal variables were summarized with median and interquartile range (IQR). Wilcoxon rank sum test was used to compare such variables for two groups. Nominal data were summarized via absolute and relative frequencies. Absolute frequencies represent the count of each category, while relative frequencies indicate the proportions of each category within the sample. Statistical testing for nominal data compared between two groups was conducted with either Barnard’s unconditional tests [35] or Pearson’s chi-squared test depending on the degrees of freedom (1, respectively > 1). 

Binary logistic regression was used to assess the probability of initiation of pharmacotherapy by OGTT glucose levels and based on risk factors such as age, pre-pregnancy BMI, parity, conception mode and history of GDM. For the predictors used in the logistic regression model, log odds were calculated. These show how a one-unit change in a predictor is associated with a change in the probability of initiating pharmacotherapy. Additionally, for the log odds, the 95% confidence intervals (CIs) were computed. Marginal effects, along with (pointwise) 95% CI bands, have been plotted either as lines in the case of numerical parameters, or as dots in the case of categorical parameters. A two-sided *p* value ≤ 0.05 was considered statistically significant. *p* values were interpreted in an explorative manner, aiming to generate new hypotheses.

## 3. Results

A total of 446 mothers with twin pregnancies were included in this study, of which 309 were dichorionic twins and 137 monochorionic twins, assessed retrospectively. Overall, the median age was 32 years and the pre-pregnancy BMI 25.71 kg/m^2^. The glucose levels at fasting reached a mean of 92 mg/dL, while the 1 h OGTT exceeded the threshold to a mean of 182 mg/dL and the 2 h OGTT slightly exceeded the threshold with a mean of 141 mg/dL. 

Of the 446 mothers, 200 (44.8%) required IT, while 246 (55.2%) were managed with lifestyle modification solely. It was found that mothers carrying a twin pregnancy and requiring pharmacotherapy were older with a median age of 33 years, had more offsprings, had a higher pre-pregnancy BMI with a median of 27.06 kg/m^2^, reached higher plasma glucose concentrations during the fasting and 1 h plasma glucose OGTT and had a higher rate of assisted reproduction treatment. If gestational diabetes occurred in a previous pregnancy, women with a subsequent twin pregnancy had a higher probability for IT in the course of their pregnancy complicated by GDM. We found a significant difference between these two groups with respect to the following characteristics: pre-gestational BMI (*p* < 0.01), fasting glucose levels (*p* < 0.01) and 60 min OGTT (*p* < 0.01), a positive history of GDM in a prior pregnancy (*p* < 0.01) and conception mode (*p* < 0.05). Additionally, there was a significant difference with respect to parity (*p* < 0.05). No association regarding the chronicity of the pregnancy (*p* > 0.05) and the OGTT of 120 min (*p* > 0.05) and use of pharmacotherapy in the pregnancy could be determined (Table 1).

Furthermore, to evaluate the need for a pharmacotherapy, a logistic regression analysis was performed with the predictors age, BMI, all time points of OGTT, a positive history of GDM and conception mode. 

**Figure 1:**
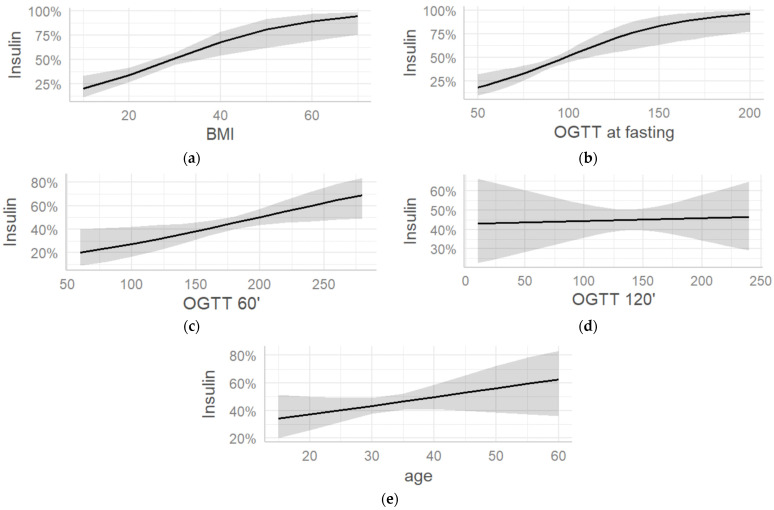
(**a**) Pre-pregnancy BMI (kg/m^2^) and the association of insulin therapy; (**b**) OGTT at fasting (G0; (mg/dL)) and the association of insulin therapy; (**c**) OGTT 60′ (60 min OGTT, G60, (mg/dL)) and the association of insulin therapy; (**d**) OGTT 120′ (120 min OGTT, G120, (mg/dL)) and the association of insulin therapy; (**e**) age and the association of insulin therapy. Marginal effects derived via logistic regression.

Logistic regression graphs demonstrate the association of BMI and the influence on insulin therapy (Figure 1a–e). The higher the BMI pre-pregnancy, the more likely it is for the twin pregnancy to be managed by pharmacotherapy to treat GDM. An obese pre-pregnancy BMI of 30 reveals a 50% chance of the affected woman to have to be treated with insulin during her course of twin pregnancy. A pre-pregnancy BMI of 25 has a 40% likelihood to need pharmacotherapy for the optimum treatment of her glucose tolerance disturbance (Figure 1a). Regarding the OGTT, a glucose measurement of 92 at fasting gives a 50% chance of necessity for insulin to treat GDM and it increases with rising OGTT values at fasting (Figure 1b). When women reach the threshold of 180 mg/dL glucose at the 60 min OGTT, the feasibility for insulin treatment is nearly 50% and increases with rising values (Figure 1c). On the contrary, we see the ratio between OGTT at 120 min and insulin therapy. When the threshold of glucose 153 mg/dL is reached, the probability of insulin therapy is 45%, and it remains rather constant with a minimal rising tendency (Figure 1d). Although age did not show a significant association with the need for pharmacotherapy, a trend with advancing age can be determined (Figure 1e).

**Figure 2:**
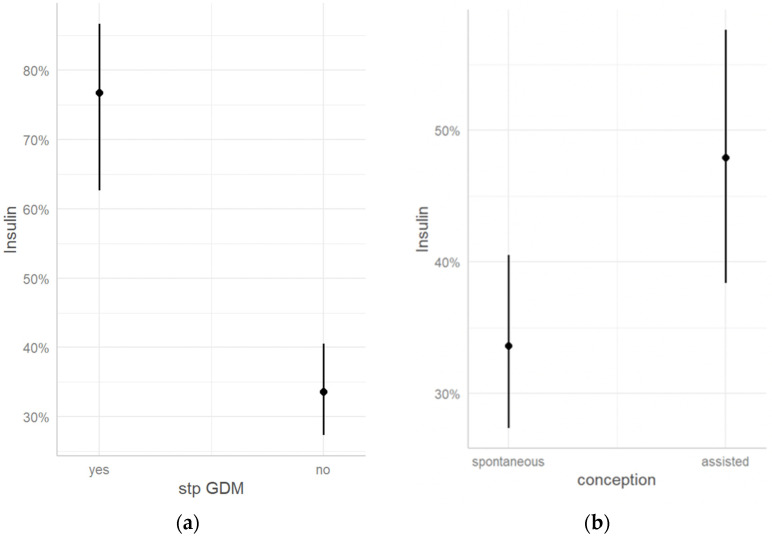
(**a**) Insulin therapy as compared to GDM in previous pregnancy (St.p. GDM); (**b**) Insulin therapy as compared to conception mode. Marginal effects derived via logistic regression. Abbreviations: St.p. = Status post.

Furthermore, individuals with a prior history of GDM exhibited a significantly greater likelihood of requiring insulin therapy during pregnancy in comparison to those without a previous GDM diagnosis (with rates exceeding 75% and 30%, respectively, as seen in Figure 2a). In cases where pregnancy resulted from assisted reproduction methods, the probability of commencing insulin treatment exceeded 45%, while patients who conceived naturally had a slightly higher than 30% chance of initiating insulin therapy (Figure 2b). Confidence intervals and a logistic regression table of the variables are visualized in Table 2.

## 4. Discussion

In this retrospective cohort study, we analyzed the need for pharmacotherapy in twin pregnancies complicated by GDM. The major finding of our study was that the need for insulin therapy was associated with a higher pre-pregnancy BMI, elevated 75 g OGTT glucose levels at fasting and 1 h, a known history of gestational diabetes in a previous pregnancy as well as multiparity and assisted reproduction when compared to gestational diabetes in twin pregnancies managed by lifestyle modification. These clinical factors show a good prediction for a pharmaceutical intervention. 

Our findings are consistent with previous research on twin pregnancies affected by gestational diabetes. In these cases, women with gestational diabetes tended to be older, have higher body weight, conceive through in-vitro fertilization, and have a history of previous pregnancies with gestational diabetes. Additionally, they were more likely to have a family history of diabetes in first or second-degree relatives compared to mothers pregnant with twins who did not have gestational diabetes [17]. 

A BMI >27 kg/m^2^ alongside with other clinical factors such as glycosuria, maternal age over 30 years, a suspected macrosomia or GDM in a prior pregnancy have shown to be the best independent predictors for GDM in singleton pregnancies according to the Austrian Gestational Diabetes Study (AGDS). Additionally, GDM was associated with a two times fold of an overweight BMI [36]. Even mothers with twin pregnancies with type 1 and type 2 diabetes diagnosed before conception were significantly more likely to be older and to have a higher BMI as compared to mothers with twin pregnancies without diabetes [37]. The findings of increased maternal age and BMI in the woman with gestational diabetes is not surprising, given that age and obesity are among the most important risk factors for the development of type 2 diabetes [38]. A body mass index above 27 kg/m^2^ is also a risk factor for the initiation of pharmacotherapy in twin pregnancy, as shown by our study. We confirm that mothers of twin pregnancies requiring intensified glucose management with insulin are heavier and reached higher glucose measurements during the OGTT.

Zhang et al. showed that an elevated fasting glucose level on the OGTT and the 2 h OGTT measurement are a strong predictor for insulin therapy in singleton pregnancies [25]. While Langer observed a significant positive correlation between fasting glucose levels exceeding 105 mg/dL and various maternal and perinatal complications, including fetal macrosomia, neonatal hypoglycemia, hypertensive syndromes and the likelihood of cesarean delivery. Consequently, Langer concluded that fasting glucose levels surpassing this threshold indicate the necessity for insulin therapy. Furthermore, the authors revealed that, among pregnant women, approximately 70% of those with fasting glucose levels at or below 95 mg/dL achieved satisfactory glycemic control through dietary control. On the other hand, only 30% of women with fasting glucose levels above 95 mg/dL achieved adequate glucose control with nutritional therapy [39]. Further studies have reported a significant relationship between fasting plasma glucose levels and the need for insulin therapy for treatment of gestational diabetes mellitus. More exactly, this relationship has been reported for fasting plasma glucose levels ≥89.5 mg/dL [40] and 87 mg/dL [41]. Of utmost importance to mention is that all of these studies were specifically conducted in the context of singleton pregnancies. We demonstrated fasting glucose levels at baseline as well as the 1 h OGTT in twin pregnancies to be elevated and of significance for insulin therapy. Meanwhile, we could not determine elevated 2 h OGTT measurements for the IT group. To the best of our knowledge, this study stands as the first investigation to look into the correlation between impaired oral glucose tolerance test results in twin pregnancies and the commencement of pharmacotherapy. Consequently, the only basis for comparison lies with singleton pregnancies that necessitated insulin treatment in the context of gestational diabetes mellitus.

It is important to mention that our data did not show any significant difference when it comes to the use of pharmacotherapy based on monochorionic or dichorionic twin pregnancies. A study suggested a higher risk of gestational diabetes in monochorionic twin pregnancies compared to dichorionic twin pregnancies [17], while another study did not find any noticeable difference [42]. When comparing the risk of gestational diabetes between twin and singleton pregnancies, it was found that the risk of gestational diabetes in twin pregnancies was reported to be of 7.7% compared to 4.1% in singleton pregnancies in one study [11], of 3.89% versus 2.32% in another study [12] and of 10.1% versus 2.9% in yet another study [13]. However, we could not confirm a higher risk of needing insulin therapy in twin pregnancies with gestational diabetes based on monochorionic or dichorionic twin pregnancies.

In terms of pharmacological management, the current data mostly address insulin therapy. Metformin may be offered to patients with GDM, especially to insulin-resistant overweight females either as monotherapy or as combined pharmaceutical intervention with insulin [43], but remain an off-label use. Therefore, we did not include Metformin therapy in our statistical analysis. 

Even within the domain of contemporary healthcare, there are still clinical questions that await comprehensive exploration, particularly concerning multiple pregnancies. When it comes to gestational diabetes mellitus, the diagnostic criteria, screening methods and treatment protocols have been transplanted directly from those designed for singleton pregnancies, a practice that has persisted over the years despite lacking a robust scientific foundation. Recognizing that twin pregnancies introduce added complexities and an elevated risk of GDM, our study makes valuable contributions. It equips us with essential tools and clinical parameters such as pre-pregnancy BMI, fasting and 1 h OGTT results, multiparity, a history of previous gestational diabetes and the role of assisted reproduction. These parameters enable us to identify patients at higher risk of requiring intensified glucose therapy and to closely monitor them in terms of pharmacotherapy. This proactive approach is aimed at reducing complications for both the expectant mothers and their unborn fetuses during pregnancy and the postpartum period.

Furthermore, the vigilant monitoring of glucose levels and the application of diverse treatment approaches elevate the overall quality of care and counseling for twin pregnancies affected by diabetes. These measures play a pivotal role in early and precise risk stratification, streamlining a timely intervention for high-risk patients.

Our findings are of further of clinical relevance, as women with pregestational diabetes pregnant with twins are significantly more likely to have an adverse pregnancy outcome compared to women with twin pregnancies without diabetes [37]. All patients must be instructed on their elevated risk to develop type 2 diabetes mellitus later in life with a reoccurring rate for GDM of 20–50% in a preceding pregnancy and a higher risk for cardiovascular disease, and these patients must especially be screened for dyslipidemia and hypertension. Therefore, these patients must be informed about diabetes prevention [44]. A reclassification of maternal glucose tolerance must take place 4 to 12 weeks after childbirth using a standard 2 h, 75 g OGTT. If postpartum prediabetes is diagnosed with impaired glucose tolerance in the 2 h OGTT (ranging from 140 to 199 mg/dL) or elevated fasting glucose (between 100 and 125 mg/dL), dietary modifications should be recommended, and an increase in physical activity, particularly endurance training, is encouraged [3]. Already during her first consultation, the pregnant female should be evaluated for her risk of developing gestational diabetes as well as for risk factors regarding her need for pharmacotherapy to achieve optimal glycemic control. 

While we offer predictive factors for twin pregnancies to evaluate and enhance the treatment algorithm, it is essential to note that further validation is required. The retrospective nature of this study limited our ability to assess various components of the “metabolic syndrome” or lifestyle habits. As a result, additional research is needed to delve deeper into these aspects and validate our findings.

### Strengths and Limitations

Our study’s limitations can be ascribed to its retrospective study design. One of the main limitations includes the exclusion of Metformin therapy, along with the unavailability of a standardized HbA1c assessment at the time of diagnosis for our patient group. It is crucial to acknowledge that HbA1c is affected by pregnancy-specific changes [45,46] and is considered a weak surrogate for insulin sensitivity and secretion. This is due to the fact that it does not offer additional information beyond the glycemic parameters measured during the oral glucose tolerance test, which contrasts with the dynamic measurements of glucose levels [47]. 

Our study shows several strengths, notably a large study population comprising 446 pregnant women with twin pregnancies. Additionally, we were able to gather substantial and reliable data, which facilitated a robust retrospective evaluation of this study.

## 5. Conclusions

Pre-pregnancy BMI as well as glucose tolerance test at fasting and 1 h glucose levels, a previous history of GDM and assisted reproduction are promising predictors of the need for insulin therapy in mothers carrying pregnancies complicated by GDM and twinning. Considering these factors can help to offer better pregnancy care and counselling in diabetic twin pregnancies, through enabling an early and accurate classification for the early treatment of high-risk patients.

## Figures and Tables

**Table 1:** Characteristics of the study population and logistic regression for initiation of insulin therapy vs. lifestyle modification in twin pregnancies with GDM.

	*n*	GDM-IT	GDM-LSM	*p*-Value
Total	446	200	246	
Age (years)	32 (29–36)	33 (30–36)	32 (29–36)	>0.05
BMI (kg/m^2^)	25.71 (22.22–30)	27.02 (23–32)	24.71 (22–29)	<0.01
Parity	1 (1–3)	2 (1–3)	1 (1–2)	<0.05
G0 (mg/dL)	92 (83–98)	94.5 (88–103)	90 (82–95)	<0.01
G60 (mg/dL)	182 (157–196)	184 (161–201)	180 (156–192)	<0.01
G120 (mg/dL)	141 (120–162)	139 (118–166)	143 (121–160)	>0.05
pGDM				<0.01
yes	73	59	14	
no	373	141	232	
Conception mode				<0.05
assisted	152	81	71	
spontaneous	294	119	758	
Chorionicity				>0.05
DC	309	141	168	
MC	137	59	78	

Note: Data are median and interquartile range (IQR) for pregnant women affected by GDM and treated with lifestyle modification (GDM-LSM) or requiring insulin therapy (GDM-IT). Age (years); BMI: pre-gestational body mass index (kg/m^2^); Parity; pGDM: previous pregnancy with gestational diabetes mellitus; G0: fasting plasma glucose (mg/dL); G60: plasma glucose 60 min after oral glucose load (mg/dL); G120: plasma glucose 120 min after oral glucose load (mg/dL). Abbreviations: *n* = number of patients; GDM-IT: gestational diabetes treated with insulin therapy; GDM-LSM: gestational diabetes mellitus treated with lifestyle modification; DC: dichorionic twins; MC: monochorionic twins. Tests performed: Wilcoxon rank sum test and Barnard’s unconditional test (numerical data and categorical data, respectively).

**Table 2:** Logistic regression table.

Predictor	Estimate	95% Confidence Interval (CI)		*p*-Value
Intercept	−4.896	−7.8134	−1.9785	0.001
Age	0.01	−0.0327	0.0519	0.6567
BMI	0.07	0.0278	0.112	0.0011
OGTT at fasting	0.031	0.0122	0.0489	0.0011
OGTT at 60′	0.01	0.0019	0.019	0.0166
OGTT at 120′	−0.001	−0.0085	0.0067	0.8130
St.p. GDM	−1.876	−2.5821	−1.1705	0.0000
Assisted conception	−0.599	−1.0859	−0.1112	0.0161

Note: Data are represented as 95% confidence interval (CI). Age (years); BMI: pre-gestational body mass index (kg/m^2^); OGTT at fasting: fasting plasma glucose levels (mg/dL); OGTT at 60′: plasma glucose 60 min after oral glucose load (mg/dL); OGTT at 120′: plasma glucose 120 min after oral glucose load (mg/dL); Abbreviations: St.p. = Status post; Assisted conception: mode of conception.

## Data Availability

The data presented in this study are available upon request from the corresponding author. The data are not publicly available due to data privacy.
